# Hyperacute Superior Vena Cava Syndrome Secondary to Central Venous Catheter-Associated Thrombosis in a Patient with Tongue Cancer: An Unusually Rapid Presentation

**DOI:** 10.7759/cureus.95058

**Published:** 2025-10-21

**Authors:** Mahmoud Elnadi, Sohaib Eladl, Sherouk Elsheiwi, Shaheryar Khan, Abubaker Mohammed

**Affiliations:** 1 Accident and Emergency, Darlington Memorial Hospital, Darlington, GBR; 2 Accident and Emergency, Blackpool Teaching Hospitals NHS Foundation Trust, Blackpool, GBR; 3 Radiology, Mansoura University, Mansoura, EGY; 4 Emergency Medicine, Darlington Memorial Hospital, Darlington, GBR

**Keywords:** central venous catheter complications, cerebral venous thrombosis, squamous cell carcinoma of the tongue, superior vena cava (svc) obstruction, total glossectomy

## Abstract

Superior vena cava (SVC) syndrome is most often caused by malignant compression or thrombus formation and typically presents with progressive symptoms over weeks to months, while even acute cases usually evolve over several hours to days. We report a rare case of hyperacute SVC obstruction in a 53-year-old man with recurrent tongue carcinoma, a Hickman line in situ, and therapeutic anticoagulation for a recent pulmonary embolism. He developed profound cyanosis, facial and upper body swelling, and transient loss of consciousness within less than an hour. Imaging revealed a central line-associated thrombus causing complete SVC occlusion, and multidisciplinary input was required to balance the risks of catheter removal versus retention. This case underscores the importance of rapid recognition and coordinated management of SVC syndrome in high-risk oncology patients with central venous access.

## Introduction

Superior vena cava (SVC) syndrome is a condition caused by obstruction of venous return through the SVC, leading to an elevated venous pressure in the upper body [1]. Malignancy, most often lung cancer or lymphoma, accounts for the majority of cases [2]. However, catheter-associated thrombosis has become an increasingly recognized etiology in oncology patients with indwelling central venous devices [3]. The classic clinical presentation includes facial swelling, upper limb edema, venous distension, and dyspnea, which typically progress insidiously over days to weeks as collateral venous circulation develops [4]. In contrast, acute SVC obstruction, usually thrombotic in origin, can present far more dramatically with cyanosis, syncope, headache, or even airway compromise. These acute cases generally evolve over several hours, but a fulminant onset within minutes is exceedingly rare [5]. We report the case of a 53-year-old male with tongue cancer who developed sudden, severe SVC obstruction secondary to central venous catheter thrombosis, manifesting as dramatic upper body cyanosis and recurrent collapse within minutes.

## Case presentation

A 53-year-old male patient with a history of recurrent squamous cell carcinoma of the tongue, previously treated with total glossectomy, total laryngectomy, and cricopharyngeal myotomy, was on a three-week cycle of chemotherapy. He had a Hickman line placed four months prior, last used two days before presentation. He was anticoagulated with therapeutic tinzaparin (18,000 units daily) following a pulmonary embolism two months earlier.

The patient, who communicated nonverbally via his phone, was reportedly well when his wife left the room. Within 15 minutes, she returned to find him collapsed on the floor, cyanosed, and unresponsive for two minutes before regaining consciousness. He suffered another brief collapse in the presence of paramedics. His wife reported that the swelling and the cyanosis were progressively getting worse.

**Figure 1:**
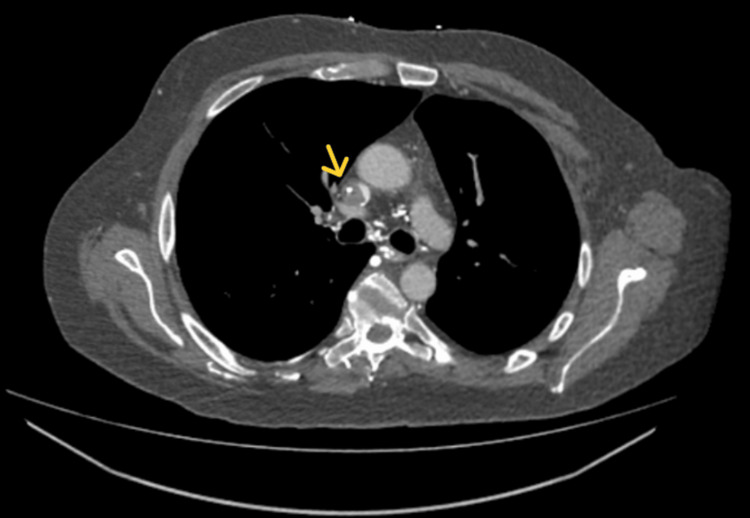
Thrombus around the distal central line, causing complete SVC occlusion SVC: Superior vena cava.

On arrival at the emergency department, he reported heaviness in the chest and head but no chest pain or shortness of breath. An examination revealed dramatic cyanosis and swelling involving the face, neck, arms, and chest down to the nipple line with distended neck veins. Objective measurements such as neck circumference or positional pulse oximetry were not obtained at the time of presentation. The description of “dramatic cyanosis and swelling” reflects the treating team’s clinical observation and examination findings. The laryngectomy tube remained patent with equal bilateral breath sounds, normal heart sounds, and hemodynamic stability. Neurological examination was unremarkable. CT chest confirmed a thrombus around the distal Hickman line, causing complete occlusion of the SVC (Figure 1).

The patient’s case was reviewed at a multidisciplinary meeting involving oncology, hematology, and vascular surgery. After discussion of risks and benefits, the plan was to continue anticoagulation and consider elective line removal once clinically stable.

## Discussion

This case demonstrates a hyperacute presentation of SVC syndrome, in sharp contrast to its more typical gradual course. Malignancy-associated SVC sydrome usually develops over weeks to months as progressive tumor growth causes extrinsic compression. In this patient, however, a catheter-associated thrombus led to a near-complete venous obstruction, with symptom onset occurring within minutes.

The abrupt deterioration underscores an important clinical consideration: in patients with central venous catheters, particularly those with underlying hypercoagulability, SVC syndrome may present as a true vascular emergency. In this instance, the patient’s extensive oncologic history, recent pulmonary embolism, and ongoing chemotherapy created a profoundly hypercoagulable state. Remarkably, the thrombus developed despite therapeutic anticoagulation with tinzaparin. An anti-Xa level measured at the time of presentation was 1.09, confirming therapeutic anticoagulation, highlighting the limitations of thromboprophylaxis in advanced malignancy.

Management was further complicated by the complex decision-making surrounding catheter retention versus removal. Removal carried the risk of thrombus mobilization and catastrophic embolization, whereas retention perpetuated obstruction and symptom burden. This scenario illustrates the complex risk-benefit analyses frequently encountered in oncologic practice. Given the risk-benefit assessment, the decision was made to remove the Hickman line electively while maintaining anticoagulation. After elective removal of the Hickman line and transition to warfarin therapy in addition to tinzaparin, the patient experienced progressive improvement, with gradual resolution of upper body swelling and cyanosis. 

Ultimately, this case emphasized the need for heightened clinical vigilance for hyperacute SVC syndrome in patients with central venous access. It also demonstrated the critical importance of a rapid, coordinated, multidisciplinary response to optimize safety and therapeutic outcomes.

Acute SVC obstruction is a rare occurrence. In 2024, Yingchoncharoen et al. described a case of malignant SVC obstruction with symptoms developing gradually over a month, not acutely as in our case [6]. Similarly, in 2022, Haider et al. reported a case of SVC syndrome due to thrombosis, which presented with a somewhat acute onset [7]. In 2023, Kanaji et al. described SVC syndrome induced by lung hyperinflation in chronic obstructive pulmonary disease, with symptoms persisting for several years [8]. In 2018, Nguyen et al. reported a case presenting with a two-month history of periorbital edema, but without an acute presentation [9].

A case of SVC syndrome caused by mediastinal lymphoma was reported in 2021 by Besteiro et al., with symptoms progressing over two weeks [10]. That same year, Joshi et al. described a combined superior and inferior vena cava syndrome due to large-cell neuroendocrine carcinoma, with symptoms progressing over four months, including dyspnea at rest, weight loss, cough, hoarseness, and hemoptysis [11]. In 2018, Hinton et al. reported a case of SVC syndrome in a patient with locally advanced lung cancer, with symptoms appearing three months after chemotherapy [12].

In 2021, Ni et al. described Hodgkin lymphoma-associated SVC syndrome with symptoms developing over three weeks [13]. A rare case of SVC obstruction in a 16-year-old boy with Burkitt’s lymphoma was reported in 2021 by Alfahadi et al., with a two-month history of neck swelling [14]. In 2016, Hassan et al. described malignant SVC syndrome presenting as syncope, with progressive neck pain and swelling over six months [15].

Another case of SVC syndrome, presenting with facial swelling, neck distension, and chest wall venous engorgement over five weeks in a patient with small-cell lung cancer on a clinical trial, was reported in 2017 by Brzezniak et al. [16]. In 2022, Rajavardhan et al. reported a rare case of SVC syndrome in a patient on veno-venous Extracorporeal Membrane Oxygenation (VV-ECMO), with symptoms developing over several days during a 14-day ECMO course [17]. Finally, Israel et al. described a case of SVC syndrome with granulomas, presenting with a 10-day history of facial swelling, dysphagia, dyspnea, and hoarseness [18].

## Conclusions

This case highlights that catheter-associated SVC syndrome may present with abrupt, severe symptoms in contrast to the more gradual course of malignancy-related SVC syndrome. It underscores the heightened thrombotic risk in oncology patients, where clinically significant thrombosis can develop despite therapeutic anticoagulation. The management challenge, whether to remove the central venous catheter or leave it in place, demonstrates the need for urgent, multidisciplinary decision-making to balance the risks of intervention against the dangers of ongoing obstruction. Ultimately, this case serves as a reminder for clinicians to maintain vigilance for this vascular emergency in any cancer patient with central venous access.
